#  Repeated Administration of Mercury Intensifies Brain Damage in Multiple Sclerosis through Mitochondrial Dysfunction

**Published:** 2016

**Authors:** Farzad Kahrizi, Ahmad Salimi, Farshid Noorbakhsh, Mehrdad Faizi, Freshteh Mehri, Parvaneh Naserzadeh, Nima Naderi, Jalal Pourahmad

**Affiliations:** a *Department of Pharmacology and Toxicology, Faculty of Pharmacy, Shahid Beheshti University of Medical Sciences, Tehran, Iran. *; b *Department of Pharmacology and Toxicology, School of Pharmacy, Ardabil University of Medical Science, Ardabil, Iran.*; c *Students research committee, School of Pharmacy, Shahid Beheshti University of Medical Sciences, Tehran, Iran *; d *Department of Immunology, Faculty of medicine, Tehran University of Medical Sciences, Tehran, Iran.*

**Keywords:** Mercury, EAE model, Brain mitochondria, Oxidative stress, Apoptosis

## Abstract

In this study we investigated the additive effect of mercury on the brain mitochondrial dysfunction in experimental autoimmune encephalomyelitis (EAE) model. Experimental animals (female C57BL/6 mice) are divided into four groups (n = 8); control, Hg, EAE, EAE with Hg. EAE model of MS induced by injecting myelin oligodendrocyte glycoprotein (MOG). Neurobehavioral alterations are recorded and then mice were sacrificed at day 28 and brain mitochondria were isolated and mitochondrial toxicity parameters including mitochondrial swelling, reactive oxygen species (ROS) formation, collapse of mitochondrial membrane potential (MMP) and cytochrome c release were measured. Our results showed that repeated treatment of mercury following induction of EAE in mice significantly increased the neurobehavioral scores, as well as mitochondrial toxicity through ROS formation, mitochondrial swelling, collapse of MMP and cytochrome c release. Our findings proved that repeated exposure with mercury accelerates progression of MS through mitochondrial damage related to oxidative stress and finally apoptosis.

## Introduction

Mercury is a heavy metal that has known as a toxicant in living organisms and exposure to mercury compounds continue to be prevalent and significant health risks to human (1). Approximately one million tons of metallic Hg has been extracted from ores during the past five centuries (2). Original routes of human exposure to Hg are environmental contaminant, industrial usage and health care. Multiple sclerosis is a chronic and progressive autoimmune disease in the central nervous system (CNS) of young adults, with over 2.5 million individuals affected worldwide (3). MS has almost unknown etiology and no effective cure at the time being. it`s treatment is supportive and symptomatic (4). Mitochondria extensive dysfunction and concomitant oxidative stress can cause many neurodegenerative diseases such as multiple sclerosis (5). There are many study that show relation between mercury exposure and MS for example mercury-containing amalgam may exert an important risk for patients with autoimmune diseases (6). Mercury concentrations in the cerebrospinal fluid and blood serum of MS patients is higher than healthy individuals (7) .It has already been demonstrated that inhibition of myelin production can be resulted from toxic effect of mercury on human oligodendrocytes (8).

In this study for evaluation of relationship progression of MS and repeated exposure of mercury we used EAE model to induce MS in C57BL/6 mice, then neurobehavioral symptoms observed and recorded. Finally We sacrificed mice at day 28 following EAE induction and then brain mitochondria were isolated to examine mitochondrial damage parameters such as mitochondrial swelling, ROS formation, collapse of MMP and cytochrome c release.

## Material and Methods

*Animals*

All experiments were performed on female C57BL/6 mice (Razi Institute, Karaj, Iran) weighing 20-25 g (12 weeks).The animals were kept under a 12 h light/dark cycle in a temperature controlled (22. ± 2 °C) environment with free access to food and water ad libitum. All studies and animal care procedures were accomplished in accordance with the local ethics committee for animal experimentation and in compliance with guidelines of Shahid Beheshti University of Medical Sciences. 

*Induction of EAE and experimental design*

EAE model for MS was induced in mice with MOG35–55 peptide emulsified in complete Freund’s adjuvant (CFA) using Hooke kits (Hooke Laboratories, Lawrence, MA, USA) according to the manufacturer’s instruction. All drugs and their respective vehicles were injected intraperitoneally. The intact (no EAE) group received CFA and pertussis toxin without MOG. Mercury sulfides suspended in carboxymethyl cellulose (CMC) then it exposed to mice oral daily (1 mg/Kg/day) for 7 days from the onset of EAE induction. (9). 

The following groups were used in this study: control group received CFA and pertussis toxin without MOG, EAE group, mercury sulfide group, and EAE + mercury sulfide group. The number of animals in each group were 8(n=8).

*Neurological assessment*

Mice were daily observed to record behavioral and neurological signs until day 28 after immunization. The signs of EAE were scored protocol of Stromnes *et al*. (10).

*Mitochondrial preparation*

At 28^th^ day after induction EAE mice were sacrificed and brain and spinal cord mitochondria were isolated using differential centrifugation (11). Mitochondria were prepared freshly for each experiment and used within 4 h of isolation. 

*Determination of mitochondrial ROS level*

The mitochondrial ROS measurement was performed using the fluorescent probe DCFH-DA. The fluorescence intensity of DCF was measured using Shimadzu RF-5000U fluorescence spectrophotometer at an excitation wavelength of 488 nm and emission wavelength of 527 nm (12, 13).

*Determination of the MMP*

Mitochondrial uptake of the cationic fluorescent dye, rhodamine123, has been used for the estimation of mitochondrial membrane potential. The mitochondrial fractions (0.5 mg protein/mL) were incubated with 10 µM of rhodamine 123 in MMP assay buffer. The fluorescence was monitored using Shimadzu RF-5000U fluorescence spectrophotometer at the excitation and emission wavelength of 490 nm and 535 nm, respectively (14, 15) .

*Determination of mitochondrial swelling*

Briefly, isolated mitochondria were suspended in swelling buffer (70 mM sucrose, 230 mM mannitol, 3 mM HEPES, 2 mM tris phosphate, 5 mM succinate and 1 µM of rotenone). The absorbance was measured at 549 nm at 15 min time intervals with an ELISA reader (Tecan, Rainbow Thermo and Austria) (13, 16). 

*Cytochrome c release assay*

The concentration of cytochrome c was determined through using the Quantikine Rat/Mouse Cytochrome c Immunoassay kit provided by R&D Systems, Inc. (Minneapolis, Minn.) according to manufacturer’s instruction (15). 

*Statistical analysis*

The results are expressed as mean ± SEM and were analyzed using Graph Pad 

Prism (version 5, Graph pad Software Inc.). A one-way or two-way analysis of variance (ANOVA) followed by Bonferroni’s tests were used for multiple comparison. For all statistical analyses, p<0.05 was considered significant.

## Results

*EAE scoring *

**Figure 1:**
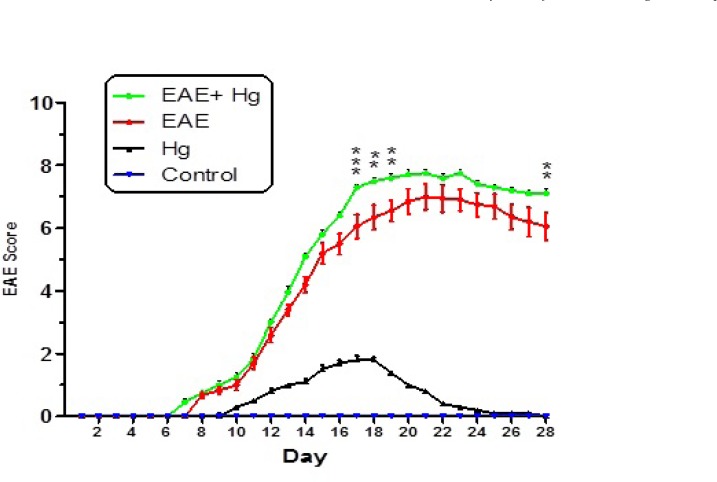
Behavioral and neurological signs in mice experimental groups. Values were presented as score of signs as described in materials and methods (n = 8). ** represents P < 0.01 significant difference, *** represents P < 0.001 significant difference, compared to EAE induced mice group.

As shown in Figure 1 behavioral and neurological signs were increased in EAE induced group which repeatedly received Hg. In the Hg^2+^treated group, there were also some behavioral and neurological signs.

*Measurement of ROS generation*

**Figure 2:**
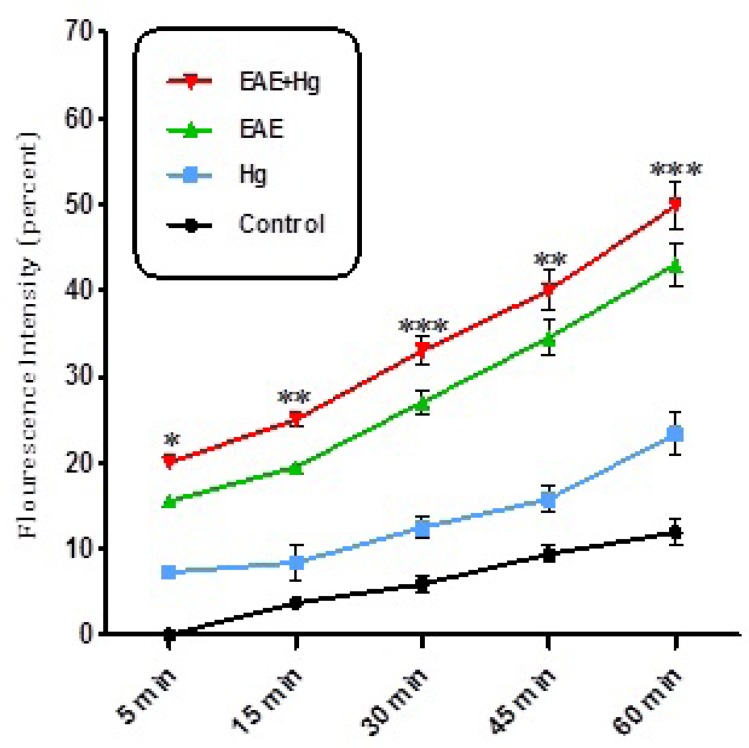
ROS formation. Exposure to mercury (Hg) intensified ROS formation in EAE + Hg group compared to EAE group. Values have been presented as percent ROS formation (n = 8). *** represents significant difference between control and EAE groups, also $$$ represents significant difference (P < 0.001) between EAE and EAE+Hg2+ groups

As shown in Figure 2. ROS significantly (P<0.05) increased in the brain mitochondria isolated from (EAE + Hg^2+^) group compared with EAE group. ROS were also significantly (P<0.05) increased in EAE group compared with control group.

*Determination of mitochondrial membrane potential*

**Figure 3:**
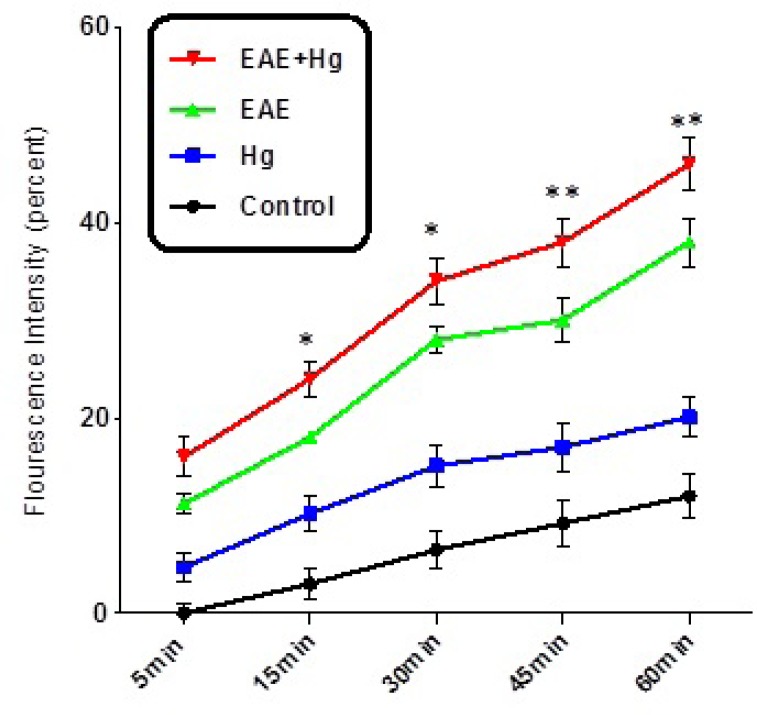
Decline of MMP. Decline of MMP significantly decreased in EAE + Hg group compared to EAE group. Values expressed as percent of MMP collapse (n = 8). *** represents significant difference between control and EAE groups, also $$$ represents significant difference (P < 0.001) between EAE and EAE + Hg groups

As shown in Figure 3. MMP significantly decreased in the brain mitochondria isolated from EAE + Hg group compared with EAE group.

*Measurement of mitochondrial swelling*

**Figure 4:**
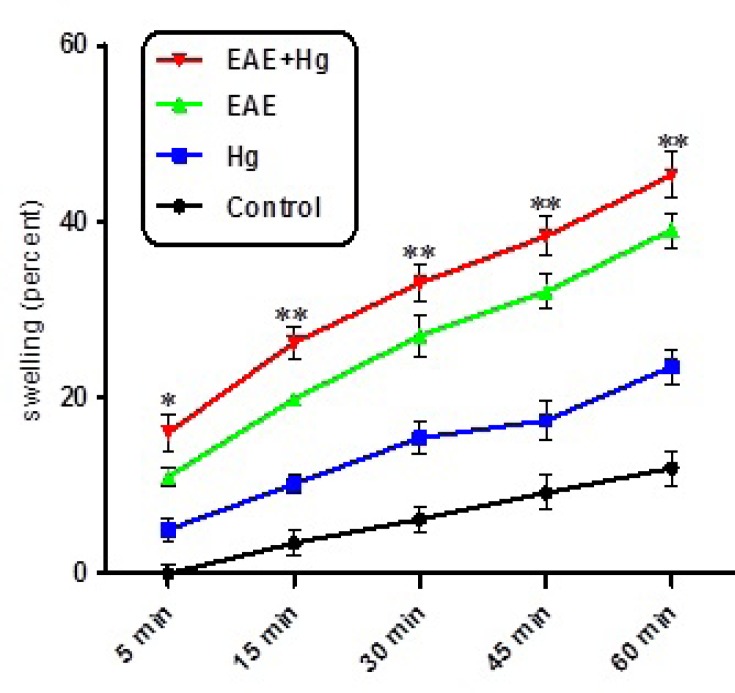
Mitochondrial swelling. Hg enhanced mitochondrial swelling in EAE + Hg group. Values have been presented as decreasing absorbance at 450 nm (n = 8). *** represents significant difference between control and EAE groups, also $$$ represents significant difference (P < 0.001) between EAE and EAE + Hg groups

As shown in Figure 4. collapse of optical absorbance at 540 nm which is consistent with mitochondrial swelling, was assayed. Our results showed that of repeated administration of mercuric sulfide significantly enhanced swelling in the brain mitochondria isolated from (EAE + Hg).

*Cytochrome c release*

**Figure 5:**
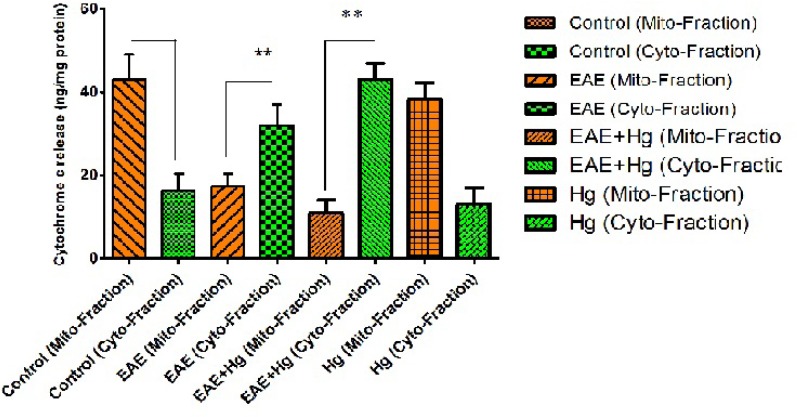
Cytochrome c release. In EAE + Hg and EAE groups cytochrome c released. Values have been presented as mean ± SD (n = 8). *** represents significant difference (P < 0. 001) compared to EAE induced group. Also $$$ represents significant difference (P < 0. 001) between EAE + Hg and EAE groups

As shown in Figure 5. there is significant enhancement in the release of cytochrome c from brain mitochondria in the (EAE + Hg) group compared with EAE group.

## Discussion

Using EAE scoring test we proved that, mercury causes behavioral, neuromuscular, sensorimotor disturbances in EAE + Hg treated mice compared to Hg group (Figure 1). Our results showed that in Hg accelerated behavioral dysfunction in EAE model. Moreover there are many previous studies that indicate relationship between MS and mercury. Mercury is a heavy metal that exerts acute and chronic toxic effects on the human body including the nervous and immune systems (17). One of the main pathways of mercury toxicity is damage to mitochondrial function (18). Malfunction of mitochondria has been associated to neurodegenerative diseases (19). The functionality of mitochondrial preparations is very useful in the study of the processes underlying pathologies of these diseases. It has already proven that use of isolated mitochondria is a very powerful tool in the study of pathologies of many neurodegenerative (20). Results of ROS measurement in isolated mitochondria showed that ROS formation was significantly increased in EAE + Hg^+2^ group (Figure 2). Following the rise of reactive oxygen species formation and consecutive oxidative damage to mitochondria, mitochondrial functions are impaired. Our findings regarding mitochondrial membrane potential collapse (Figure 3.) and mitochondrial swelling (Figure 4.) in EAE + Hg group proved this hypothesis. Previous studies with mercury have also shown increased damage through ROS formation. Our results suggest in this animal model, substantial ROS produced by mercury increases and accelerates neural damage compared to EAE group. The increased mitochondrial ROS formation can cause oxidation of a lipid membrane which consequently results in disruption of the mitochondrial membrane and subsequently the collapse of mitochondrial membrane potential (MMP) and cytochrome c release. (21). A number of studies have reported mitochondrial defects in MS. We also showed that in EAE model mitochondrial damages increased which with repeated exposure with mercury these damage significantly increased. 

Our results provided evidence that cumulative mitochondrial damages result in mercury accelerates progression MS through mitochondrial ROS formation that lead to collapse MMP, mitochondrial swelling and finally initiation of apoptosis signaling through mitochondrial pathway. To our knowledge this the first report that provides relationship between mitochondrial events and progression of MS through apoptosis by mercury in brain.

## References

[1] Gochfeld M (2003). Cases of mercury exposure, bioavailability, and absorption. Ecotoxicol. Environ. Saf.

[2] Hylander LD, Meili M (2003). 500 years of mercury production: Global annual inventory by region until 2000 and associated emissions. Sci. Total Environ.

[3] Moreau T, Coles A, Wing M, Isaacs J, Hale G, Waldmann H, Compston A (1996). Transient increase in symptoms associated with cytokine release in patients with multiple sclerosis. Brain.

[4] Rietberg MB, Brooks D, Uitdehaag BM, Kwakkel G (2004). Exercise therapy for multiple sclerosis. The Cochrane Library.

[5] Beal M (1996). Aging, energy, and oxidative stress in neurodegenerative diseases. Restor. Neurol. Neurosci.

[6] Prochazkova J, Sterzl I, Kucerova H, Bartova J, Stejskal VD (2004). The beneficial effect of amalgam replacement on health in patients with autoimmunity. Neuro Endocrinol. Lett.

[7] Attar AM, Kharkhaneh A, Etemadifar M, Keyhanian K, Davoudi V, Saadatnia M (2012). Serum mercury level and multiple sclerosis. Biol. Trace Elem. Res.

[8] Issa Y, Watts D, Duxbury A, Brunton P, Watson M, Waters C (2003). Mercuric chloride: Toxicity and apoptosis in a human oligodendroglial cell line mo3. 13. Biomaterials.

[9] Chuu J-J, Hsu C-J, Lin-Shiau S-Y (2001). Abnormal auditory brainstem responses for mice treated with mercurial compounds: Involvement of excessive nitric oxide. Toxicology.

[10] Stromnes IM, Goverman JM (2006). Active induction of experimental allergic encephalomyelitis. Nat. Protoc.

[11] Shaki F, Hosseini M-J, Ghazi-Khansari M, Pourahmad J (2013). Depleted uranium induces disruption of energy homeostasis and oxidative stress in isolated rat brain mitochondria. Metallomics.

[12] Babincova M, Bacova Z, Machova E, Kogan G (2002). Antioxidant properties of carboxymethyl glucan: Comparative analysis. J. Med. Food.

[13] Faizi M, Salimi A, Rasoulzadeh M, Naserzadeh P, Pourahmad J (2014). Schizophrenia induces oxidative stress and cytochrome c release in isolated rat brain mitochondria: A possible pathway for induction of apoptosis and neurodegeneration. Iran. J. Pharm. Res.

[14] Babincová M, Machová E, Kogan G (1999). Carboxymethylated glucan inhibits lipid peroxidation in liposomes. Zeitschrift für Naturforschung C.

[15] Talari M, Seydi E, Salimi A, Mohsenifar Z, Kamalinejad M, Pourahmad J (2014). Dracocephalum: Novel anticancer plant acting on liver cancer cell mitochondria. BioMed Res. Int.

[16] Pourahmad J, Faizi M, Abarghuyi S, Salimi A, Nasoohi S (2016). A search for mitochondrial damage in alzheimer’s disease using isolated rat brain mitochondria. Iran. J. Pharm. Res.

[17] Taueg C, Sanfilippo D, Rowens B, Szejda J, Hesse J (1992). Acute and chronic poisoning from residential exposures to elemental mercury-michigan, 1989–1990. J. Toxicol. Clin. Toxicol.

[18] Kern JK, Geier DA, Audhya T, King PG, Sykes LK, Geier MR (2011). Evidence of parallels between mercury intoxication and the brain pathology in autism. ‎Acta Neurobiol. Exp.

[19] Enríquez JA, Fernández-Sílva P, Montoya J (1999). Autonomous regulation in mammalian mitochondrial DNA transcription. Biol. Chem.

[20] Beal MF (1995). Aging, energy, and oxidative stress in neurodegenerative diseases. Ann. Neurol.

[21] Kowaltowski AJ, Castilho RF, Vercesi AE (2001). Mitochondrial permeability transition and oxidative stress. FEBS Lett.

